# CICIoT2023: A Real-Time Dataset and Benchmark for Large-Scale Attacks in IoT Environment

**DOI:** 10.3390/s23135941

**Published:** 2023-06-26

**Authors:** Euclides Carlos Pinto Neto, Sajjad Dadkhah, Raphael Ferreira, Alireza Zohourian, Rongxing Lu, Ali A. Ghorbani

**Affiliations:** Faculty of Computer Science, University of New Brunswick (UnB), Fredericton, NB E3B 5A3, Canada; e.neto@unb.ca (E.C.P.N.); raphael.ferreira@unb.ca (R.F.); alireza.zohourian@unb.ca (A.Z.); rlu1@unb.ca (R.L.); ghorbani@unb.ca (A.A.G.)

**Keywords:** Internet of Things (IoT), dataset, security, machine learning, deep learning, DoS, DDoS, reconnaissance, web attacks, brute force, spoofing, Mirai

## Abstract

Nowadays, the Internet of Things (IoT) concept plays a pivotal role in society and brings new capabilities to different industries. The number of IoT solutions in areas such as transportation and healthcare is increasing and new services are under development. In the last decade, society has experienced a drastic increase in IoT connections. In fact, IoT connections will increase in the next few years across different areas. Conversely, several challenges still need to be faced to enable efficient and secure operations (e.g., interoperability, security, and standards). Furthermore, although efforts have been made to produce datasets composed of attacks against IoT devices, several possible attacks are not considered. Most existing efforts do not consider an extensive network topology with real IoT devices. The main goal of this research is to propose a novel and extensive IoT attack dataset to foster the development of security analytics applications in real IoT operations. To accomplish this, 33 attacks are executed in an IoT topology composed of 105 devices. These attacks are classified into seven categories, namely DDoS, DoS, Recon, Web-based, brute force, spoofing, and Mirai. Finally, all attacks are executed by malicious IoT devices targeting other IoT devices. The dataset is available on the CIC Dataset website.

## 1. Introduction

Nowadays, the Internet of Things (IoT) plays a pivotal role in society and brings new capabilities to different industries [1,2,3]. IoT projects in areas such as transportation and healthcare are becoming increasingly popular, and new applications are under development [4,5]. This new paradigm relies on an extensively connected sensors and actuators network with multiple devices producing network traffic [6,7,8]. Research and industrial communities have been evolving this concept for years, and these devices are becoming more present in our daily lives [9,10,11].

Several areas have been transformed by this technology. For example, in healthcare applications, patients can be regularly monitored using IoT technology [12,13,14]. In transportation, IoT devices have been used to detect and prevent accidents [15,16,17]. Industrial IoT (IIoT) has also brought different solutions, such as high reliability and low latency automated monitoring and collaborative control [18]. IoT applications have also been developed for areas such as education [19], aviation [20], and forestry [21]. In the last decade, society has experienced a drastic increase in IoT connections [22]. In fact, IoT connections will increase in the next few years across different areas [23]. This motivates the creation and development of business ideas and new concepts that rely on a highly distributed infrastructure. In addition, various strategies have been proposed to solve potential problems in IoT operations, i.e., the deployment of new services is leveraged by the scientific findings achieved in the past few years.

Conversely, despite these benefits, several challenges still need to be faced to enable efficient and secure operations (e.g., interoperability, security, standards, and server technologies) [24,25,26,27]. The development of new applications may also bring new requirements to the systems [28,29]. For example, the Internet of Vehicles (IoV) may require more restrictive response times than common IoT applications. Furthermore, detecting and mitigating attacks performed against IoT devices is challenging due to several factors. For example, distributed connections and light devices without security mechanisms may harden the process of detecting and mitigating attacks [30,31,32,33].

Furthermore, although efforts have been made to produce datasets composed of attacks against IoT devices, several possible attacks are not considered. In addition, most efforts do not consider an extensive network topology with real IoT devices. Finally, the attacks performed against IoT devices are executed by computer systems (i.e., non-IoT devices), highlighting the need for a dataset composed of attacks performed by malicious IoT devices. To enable the development of security analytics solutions for intrusion detection in real-world scenarios, the data produced need to (i) include a variety of attacks that can harm IoT operations, (ii) be collected from an extensive topology with real IoT devices of different types and brands, and (iii) include attacks performed by malicious IoT devices.

The main goal of this research is to propose a novel and extensive IoT attack dataset to foster the development of security analytics applications in real IoT operations. To accomplish this, 33 attacks are executed in an IoT topology composed of 105 devices. These attacks are classified into seven categories, namely DDoS, DoS, Recon, Web-based, brute force, spoofing, and Mirai. In addition, all attacks are executed by malicious IoT devices targeting other IoT devices. This dataset includes multiple attacks not available in other IoT datasets and enables IoT professionals to develop new security analytics solutions. Furthermore, the data are available in different formats, allowing researchers to use features extracted in our evaluation or engineer new features.

The main contributions of this research are:

- We design a new realistic IoT attack dataset, CICIoT2023, using an extensive topology composed of several real IoT devices acting as either attackers or victims;
- We perform, document, and collect data from 33 attacks divided into 7 classes against IoT devices and demonstrated how they can be reproduced;
- We evaluate the performance of machine and deep learning algorithms using the CICIoT2023 dataset to classify and detect IoT network traffic as malicious or benign.

This paper is organized as follows: Section 2 presents an extensive comparison of the contributions of this research with other works present in the literature. Secondly, Section 3 introduces the CICIoT2023 dataset and presents the steps involved in the data collection. After that, Section 4 presents the feature extraction process and describes the data. Section 5 presents the machine learning (ML) evaluation in the classification of different attacks using the CICIoT2023 dataset. Finally, Section 6 presents the conclusion of this research.

## 2. Related Works

In the past few years, different contributions have been published regarding IoT security datasets. In fact, data have been produced with different goals and using different methods and resources. To better understand the characteristics of existing datasets, we review several initiatives present in the literature and compare them with the proposed CICIoT2023. The authors in [34] propose a novel network-based dataset for detecting botnet attacks in the IoT environment called N-BaioT (2018). Mirai and BASHLITE botnets were used to attack nine commercial IoT devices. Multiple features were extracted from the network traffic and used by a deep-learning autoencoder for attack detection. In [35], the authors introduce a host-based IoT dataset composed of data from real IoT devices. This dataset, called IoTHIDS (2018), is produced based on experiments considering a topology of three devices infected by Mirai, Hajime, Adira, BASHLITE, Doflo, Tsunami, and Wroba malware botnets.

IoT-SH (2019) [36] is a dataset composed of captures of twelve attacks (categorized into four classes) against eight different smart home devices. A three-layer Intrusion Detection System (IDS) is used considering various combinations of rule-based and machine learning approaches to classify the attacks. BoT-Iot (2019) is introduced in [37] as a realistic traffic dataset, produced considering heterogeneous network profiles. Multiple attacks are performed (e.g., DDoS, DoS, data theft, and scan) against five devices. In the evaluation process, a set of new features are selected and used based on correlation coefficient and joint entropy techniques. Various machine and deep learning models are trained to evaluate the attack detection accuracy.

The authors in [38] introduce the Kitsune (2019) dataset, which is composed of four different categories of attacks executed against nine IoT devices. In the experiments conducted, a security camera was infected by a real Mirai botnet sample. This dataset is intended to support the development of plug-and-play Network Intrusion Detection Systems (NIDS) to detect normal and malicious traffic. Similarly, IoTNIDS (2019) [39] represent an initiative focused on collecting data from a real-world IoT networking environment based on the interaction between two IoT devices (speaker and camera). Multiple attacks are analyzed in this effort, e.g., Mirai, MITM, DoS, and scanning. MedBIoT (2020) [40] is an IoT network architecture dataset based on using real and emulated devices. The authors evaluated multiple machine learning techniques using 100 statistical features extracted from the IoT network traffic. In [41], the authors propose the IoT-23 (2020) dataset. This contribution refers to a botnet dataset captured composed of real network environment captures of benign and malicious traffic.

IoTIDs (2020) [42] is proposed as a dataset composed of IoT-related flow-based features, selected and ranked by the correlation coefficients technique and the Shapiro–Wilk algorithm, respectively. In the experiments, the authors performed four different attacks against two IoT devices (speaker and camera) and recorded the data. Multiple machine learning methods were used in the evaluation process (e.g., SVM, G-NB, LDA, and LR) focusing on attack detection and classification. The authors in [43] present the MQTT (2020) dataset with the primary goal of providing realistic data that include a protocol dedicated to IoT network scenarios. Furthermore, eight IoT devices were connected to the MQTT broker and a set of 33 different features were extracted and provided to various machine learning algorithms. Similarly, MQTT-IoT-IDS (2020) [44] is another contribution focused on producing a dataset using a lightweight protocol, i.e., MQTT, which is used in IoT networks. The authors focus on replicating a realistic IoT network by using a camera feed, twelve MQTT sensors, and a broker. Five scenarios are considered based on the variation in the attacks performed. Several packet-based, uni-, and bi-flow features are used alongside six different machine learning algorithms in the evaluation phase.

In [45], the authors proposed a new telemetry-based data-driven IoT/IIoT dataset called TON-IoT (2020). This heterogeneous dataset comprises both normal and attack samples captured in different scenarios. Targeting the development of a realistic dataset, the authors include attack sub-categories, data recorded from operating system logs, and network traffic. Several machine learning and deep learning algorithms are used in the evaluation phase and the achieved results are reported in detail. Finally, the Edge-IIoTSet (2022) dataset is introduced as a realistic cybersecurity resource for IoT and IIoT applications to enable the development of Intrusion Detection Systems (IDS) in centralized and distributed applications [46]. Throughout the paper, an in-depth description of the testbed used is presented. In addition, the authors also describe the dataset generation framework. Regarding the machine learning evaluation process, centralized and federated learning considerations are presented.

## 3. The Proposed CICIoT2023

This section introduces the CICIot2023 dataset. We aim to present an in-depth description of all steps and resources involved in producing this dataset. First, we describe the CIC IoT Lab. Then, we focus on the IoT topology, listing all IoT and network devices used and how they are connected. Then, we present a discussion on all attacks that have been executed. Finally, we provide insights into how the data were collected for benign and malicious scenarios.

### 3.1. IoT Lab

The production of IoT security data that can be used to support real applications is challenging for several reasons. One of the main problems is having an extensive network composed of several real IoT devices, similar to topologies of real IoT applications. Many works adopt simulated or very few IoT devices due to costs, network equipment required (e.g., switches, routers, and network tap), and personnel dedicated to maintaining such an infrastructure.

Thereupon, the Canadian Institute for Cybersecurity (CIC) has a distinguished presence in the cybersecurity ecosystem and a history of high-impact contributions to industry and academia. Examples are datasets used to develop new cybersecurity applications and several partnerships with the industry to improve the cybersecurity practice and develop new solutions. This success enabled CIC to establish an IoT lab with a dedicated network to foster the development of IoT security solutions. In fact, by sharing the data collected from this extensive topology, we intend to foster the advancement of IoT security research and support several initiatives in different IoT security aspects.

**Figure 1:**
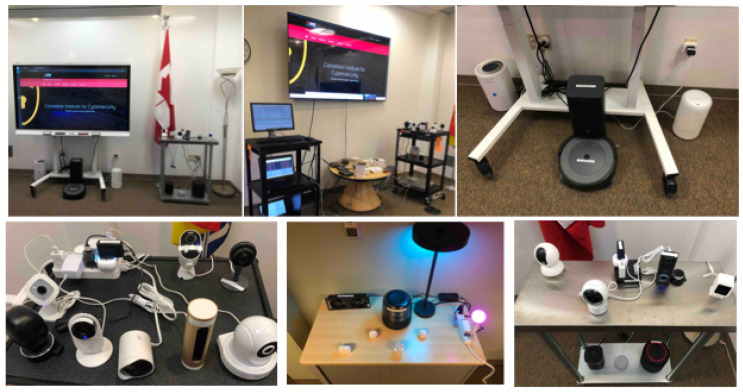
CIC IoT Lab.

Figure 1 shows the IoT lab at the CIC and its devices. Indeed, IoT devices are distributed across the lab, in which some of them are placed on the table, others on the floor, and some on the walls. We adopt a local network topology and several power plugs are available in the lab. Additionally, there are racks and storage rooms in order to organize the IoT and network devices.

### 3.2. IoT Topology

**Figure 2:**
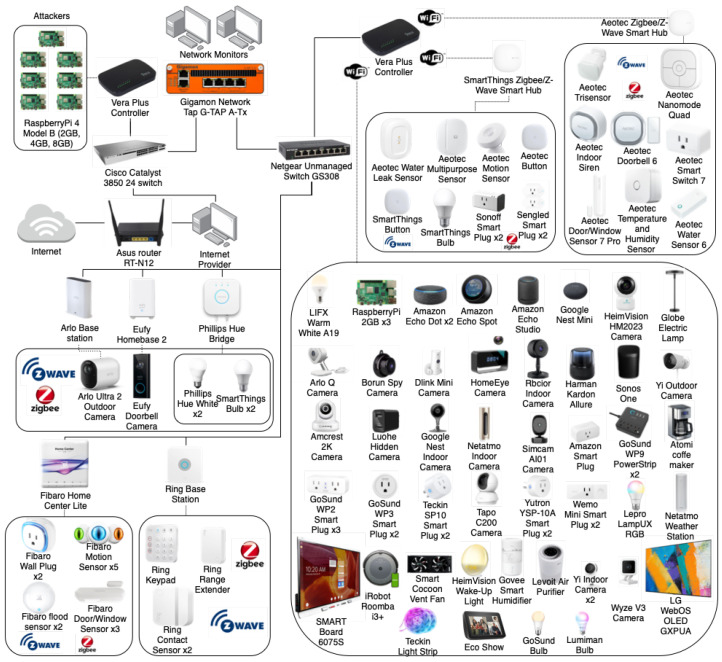
IoT network topology used in the experiments.

The IoT topology deployed to produce the CICIoT2023 is illustrated in Figure 2 and comprises 105 IoT devices. A total of 67 IoT devices were directly involved in the attacks and other 38 Zigbee and Z-Wave devices were connected to five hubs.

This topology mimics a real-world deployment of IoT products and services in a smart home environment. The devices list includes smart home devices, cameras, sensors, and micro-controllers which are connected and configured to enable the execution of several attacks and capture the corresponding attack traffic. The lab is also equipped with various tools and software, which enable us to perform several attacks and capture both benign and malicious attack traffic.

This topology is divided into two parts. In the first part, an ASUS router connects the network to the Internet and a Windows 10 Desktop computer shares this connectivity. In addition, a Cisco switch is placed between this computer and a VeraPlus access point connecting 7 Raspberry Pi devices. These devices are responsible for executing the attacks and malicious activities in the experiments. Using IoT devices as malicious agents is a CICIoT2023 characteristic not found in other efforts. Then, the Cisco switch is connected to the second part through a Gigamon Network Tap. This network device collects all the IoT traffic and sends it to two network monitors, which are responsible for storing the traffic using wireshark [47]. In fact, a network tap is a hardware device that allows for monitoring and analyzing network traffic by connecting to a network cable and providing a copy of the traffic to other monitoring and security tools. Network taps are connected in a way so as not to affect the normal operation and provide a full-duplex, non-intrusive, and passive way of accessing network traffic, without introducing any latency or affecting the performance of the network. This device has two network and two monitoring ports and is placed between the attacking and legitimate devices, connecting one port to the attackers and the other to the victim networks. Using the monitor ports, we are able to capture the traffic to and from the IoT network.

In the second part, a Netgear Unmanaged Switch is connected to five gateways and base stations to enable communication with IoT devices with protocols such as Zigbee and Z-Wave. Furthermore, another VeraPlus controller is connected to the switch. This controller is also connected to other two Zigbee/Z-Wave hubs and to several devices considered victims in the attacks performed. The list of all IoT devices used in this dataset is presented in Table 1. Note that Zigbee and Z-wave devices do not have a MAC address and are labeled as “Not Applicable” (N/A) for that particular column.

### 3.3. Data Collection of Benign and Malicious Scenarios

As described in Section 3.2, a network tap and two traffic monitors are dedicated to monitoring the network traffic. Every packet sent through the network is stored in separate computers. In fact, the network has two different interfaces, which are associated with two other monitoring ports that send incoming packets to these computers. Hence, the network traffic is monitored using Wireshark [47] and stored in pcap format. Since two data streams are stored, mergecap [48] is used to unify pcap files for each experiment.

**Figure 3:**
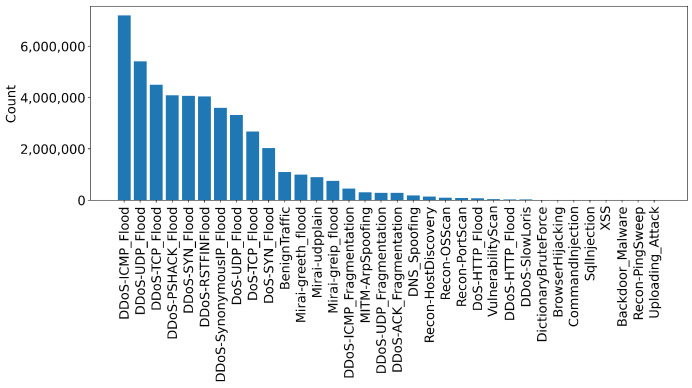
Number of rows for each scenario.

**Figure 4:**
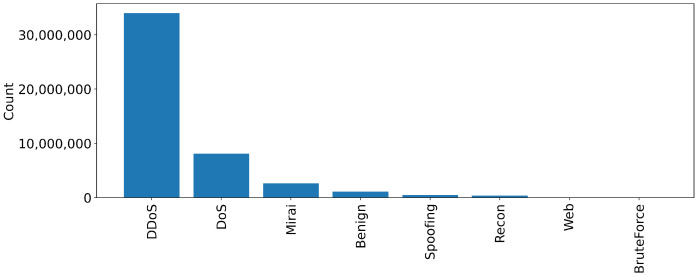
Number of rows for each category.

For each attack, a different experiment is performed targeting all applicable devices. In all scenarios, the attacks are performed by malicious IoT devices targeting vulnerable IoT devices. For example, DDoS attacks are executed against all devices, whereas web-based attacks target devices that support web applications. Table 2 depicts the tools used to perform all attacks alongside the number of rows generated. In addition, Figure 3 and Figure 4 illustrate the instances count for each attack and category. The values are also presented in Table 3.

#### 3.3.1. Benign Data Generation

The benign data represent the legitimate use of the IoT network. In this sense, the main goal of the data-capturing procedure relies on gathering IoT traffic in idle states and with human interactions (e.g., sensor data, echo dot requests, and accessing video feeds from smart cameras).

In terms of hardware for capturing, we relied on a network tap combined with two network monitors. In terms of software used, we adopted Wireshark to capture the entire traffic. Furthermore, all IoT devices are configured with default parameters and without malicious or attacking scripts. In this sense, benign data traffic gathering happens when there are no attacks. This process was conducted over a period of 16 h.

#### 3.3.2. Executing DoS and DDoS Attacks

These attacks refer to flooding threats to compromise the availability of IoT operations. In the case of Denial-of-Service (DoS) attacks, one Raspberry Pi is responsible for flooding IoT devices. Furthermore, multiple Raspberry Pis are used to execute Distributed Denial-of-Service (DDoS) attacks through an SSH-based master-client configuration. The attacks executed are:

- **ACK Fragmentation:** a relatively small number of maximum-sized packets is used to compromise the network operation. In many cases, these fragmented packets are successfully sent and handled by routers, firewalls, and intrusion prevention systems, given that fragmented packets recompilation is not performed [62];
- **Slowloris:** relies on using partial HTTP requests via open connections to a targeted Web server focusing on the application layer [63];
- **ICMP/HTTP/UDP/TCP Flood:** based on overwhelming a targeted device with different packet types [64,65,66];
- **RST-FIN Flood:** degrades networking capabilities by forwarding continuously RST-FIN packets towards a specific target [67];
- **PSH-ACK Flood:** degrades server operation by flooding using PUSH and ACK requests [68];
- **UDP Fragmentation:** refers to a special UDP flood that consumes more bandwidth while reducing the number of packets [69];
- **ICMP Fragmentation:** relies on the use of identical fragmented IP packets containing a portion of a fragmented ICMP message [70];
- **SYN Flood:** is a specific type of TCP flood that targets the initial handshake of the TCP connection. The SYN flood sends a large number of SYN (synchronize) packets to the targeted server, but it never completes the handshake by sending the final ACK (acknowledge) packet [71];
- **Synonymous IP Flood:** an extensive number of manipulated TCP-SYN packets with source and destination addresses as the targeted address, which leads the server to use its resources to process the incoming traffic [72].

#### 3.3.3. Gathering Information from the IoT Topology

These attacks gather all possible information about the target. In addition, an attacker can use a reconnaissance (i.e., scan) attack as a preparation step for other attacks. There are multiple ways to perform these attacks, and some of the most popular and threatening variations are:

- **Ping Sweep:** A ping sweep attack, also known as a ping scan, is a type of reconnaissance attack used to identify active hosts on a network. It involves sending a series of ICMP (Internet Control Message Protocol) Echo Request (ping) packets to a range of IP addresses on a network, and then analyzing the ICMP Echo Reply (pong) packets that are returned to identify which hosts are active and responding [73];
- **OS Scan:** An OS (operating system) scan attack, also known as an operating system fingerprinting attack, is a type of reconnaissance attack that is used to identify the type and version of an operating system running on a targeted host. The attacker uses various techniques to gather information about the targeted host, such as analyzing the responses to network packets, or examining the behavior of open ports and services, in order to determine the type and version of the operating system [74];
- **Vulnerability Scan:** A vulnerability scan attack is a type of network security assessment that involves automated tools to identify potential vulnerabilities in a computer system or network. The goal of a vulnerability scan is to identify security weaknesses that could be exploited by an attacker to gain unauthorized access to a system or steal sensitive information [75];
- **Port Scan:** A port scan attack is a type of reconnaissance attack that is used to identify open and active ports on a targeted host. The attacker sends a series of packets to various ports on the targeted host, attempting to establish a connection. The responses to these packets are then analyzed to determine which ports are open, closed, or filtered [76].
- **Host Discovery:** A host discovery attack, also known as a host identification or host enumeration attack, is a type of reconnaissance attack that is used to identify active hosts on a network. It involves using various techniques to identify the IP addresses of devices that are connected to a network, and it is the first step in many cyber-attacks [77].

#### 3.3.4. Exploiting Web-Based Vulnerabilities

When executing these attacks, web services running on IoT devices were targeted. Web-based attacks are concerned with targeting web services in several ways. These attack types include injection, hijacking, poisoning, spoofing, and DoS [78]. The web-based attacks executed in this research are:

- **SQL Injection:** an attack that targets web applications by injecting malicious SQL code into the application’s input fields. The goal of an SQL injection attack is to gain unauthorized access to a database, steal sensitive information, or execute arbitrary commands on the database server [79];
- **Command Injection:** an attack that targets web applications by injecting malicious commands into an input field with the ultimate goal of gaining unauthorized access to a system, stealing sensitive information, or executing arbitrary commands on the targeted system [80];
- **Backdoor Malware:** involves installing malware on a targeted system that allows the attacker to gain unauthorized access to the system at a later time. The malware, known as a “backdoor,” creates a hidden entry point into the system that can be used to bypass security measures and gain access to sensitive information or perform malicious actions [81];
- **Uploading Attack:** targets a web application by exploiting vulnerabilities in the application’s file upload functionality. The goal of an uploading attack is to upload malicious files, such as malware, to a targeted system and use them to gain unauthorized access or execute arbitrary code on the targeted system;
- **Cross-Site Scripting (XSS):** allows an attacker to inject malicious code (e.g., a script) into a web page. The injected script can then be executed by the web browser of any user with access to the page, allowing the attacker to steal sensitive information (e.g., cookies, session tokens, and personal data) or to perform other malicious activities (e.g., traffic redirection) [82];
- **Browser Hijacking:** a type of cyber attack in which an attacker modifies a web browser’s settings, such as the home page, default search engine, or bookmarks in order to redirect the user to a different website or display unwanted ads. The goal of a browser hijacking attack is to generate revenue through advertising or to steal personal information [83].

#### 3.3.5. Spoofing Communication

Spoofing attacks enable malicious actors to operate under the identity of a victim system and gain illegitimate access to the network traffic. The main focus of such a procedure includes gaining access to systems, stealing data, and spreading malware [84]. Two of the most popular spoofing attacks are:

- **ARP spoofing:** relies on the transmission of manipulated ARP (Address Resolution Protocol) messages to associate the MAC address of the malicious device with the IP address of some other legitimate device in the network. This enables attackers to intercept, modify, or block network traffic [85];
- **DNS spoofing:** relies on the alteration of DNS entries in a DNS server’s cache, redirecting users to manipulated or malicious websites. This enables attackers to steal sensitive information, spread malware, and perform other malicious actions [86].

#### 3.3.6. Brute-Force Threats

Brute-force attacks consist of the submission of data (e.g., passwords or passphrases) to eventually gain access to systems [87]. Among the several procedures that can be executed, a dictionary brute-force attack is a type of attack that attempts to guess a password or passphrase by repeatedly trying words from a pre-defined list of words obtained from various sources. The goal of the attack is to find the correct password by trying all the words in the dictionary [88].

#### 3.3.7. Mirai as an IoT Threat

**Figure 5:**
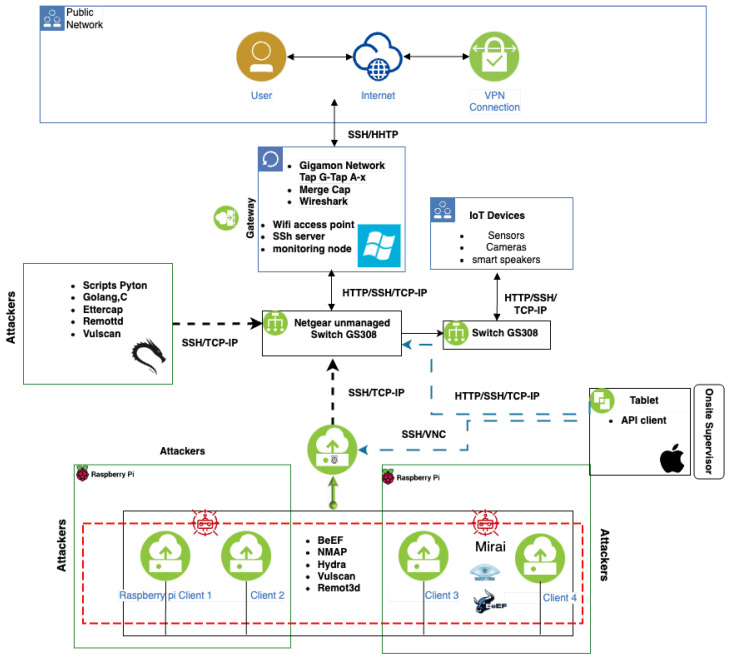
Basic attack framework for the dataset.

The Mirai attack is a large-scale DDoS that can target IoT devices. In this paper, we are conducting different variations of Mirai attacks by using five different raspberries, as illustrated in Figure 5, alongside the connections considered in the different IoT network layers. In order to connect to the Internet, a gateway uses a Windows 10 instance to provide and monitor Internet access. This access is possible through a Netgear unmanaged switch that connects attackers and general IoT devices. Several tools are used to perform the attacks and a special Mirai configuration is also adopted. An online IoT supervisor coordinates the operation of the multiple IoT devices in the topology (e.g., sensors, cameras, and smart speakers). Finally, some other works do not consider Mirai in their attack set. In fact, we focus on several attacks that can be executed against IoT devices, and we consider the analysis and execution of new IoT attacks in the future directions of this research (e.g., attacks using future protocols).

This attack infected devices to form a botnet that can flood targeted victims. This threat can cause disruption in different contexts and some of its most popular variations are:

- **GREIP:** Within the GRE packet, this attack floods the target system with encapsulated packets. The internal data comprise random IPs and ports, whereas the external layer contains actual IPs [89];
- **GREETH:** This attack presents a similar procedure to GREIP. However, the main focus is on the packet encapsulation approach, which is based on the ethernet header [89];
- **UDP Plain:** This threat focuses on flooding targeted victim systems with UDP packets considering a repeated packet segment. However, the payload sent is different for each packet [89].

## 4. Feature Extraction and Data Description

The CICIoT2023 dataset is available in two different file formats: pcap and csv. Pcap files comprise the original data generated and collected in the CIC IoT network in different scenarios. These files contain all packets sent and can be used to extract and engineer other features. Furthermore, csv files present a simpler way of loading and using the data. Those files are composed of features extracted from the original pcap files summarized by a fixed-size packet window. In other words, the features are extracted from a sequence of packets carrying information between two hosts.

**Figure 6:**
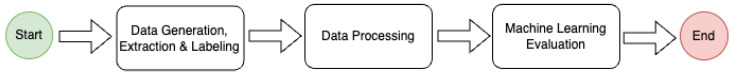
Method adopted to produce the dataset.

The method adopted to produce the dataset is illustrated in Figure 6. Firstly, the data are generated (i.e., captured), extracted, and labeled. This refers to the initial step, in which the actual attacks are executed against IoT devices. Then, the data are processed in a way to enable researchers to access the data generated easily. Finally, we conduct a machine learning (ML) evaluation to show how classification capabilities can be leveraged by the proposed dataset.

**Figure 7:**
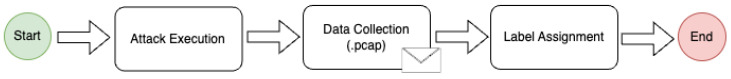
Method adopted to produce the dataset.

Figure 7 illustrates how the data generation, extraction, and labeling are conducted for each attack scenario (and benign scenario). The first phase relies on the use of different tools presented in Table 2 to execute attacks against IoT devices in the network. After that, the network traffic is captured in pcap format using Wireshark. Finally, for each attack executed, the entire traffic captured is labeled as belonging to that particular attack.

**Figure 8:**
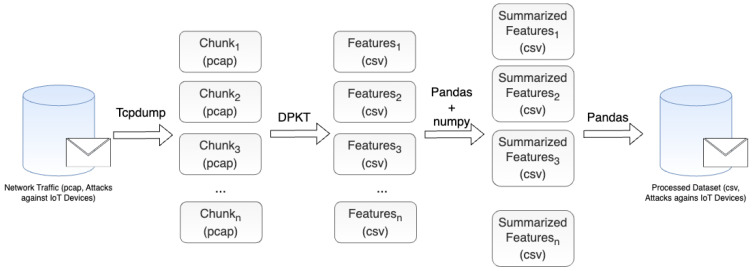
Data processing: converting pcap files to csv.

Regarding the data processing step, illustrated in Figure 8, the network traffic data composed of captures of all attacks alongside benign traffic are used. As it represents about 548 GB worth of traffic data, we split it into smaller chunks of 10 MB to perform the conversion in parallel. This process is conducted using TCPDUMP [90]. After that, a parallel procedure is executed to extract several features using the DPKT package [91] and store them in separate csv files. These features are described in Table 4. In this process, DPKT is used to enable a flexible feature extraction procedure considering important attributes of the IoT operation highlighted in previous works. Conversely, other tools can also be used to extract features, e.g., CICFlowMeter [92] and Nfstream [93]. In this stage, we also perform the data cleaning by removing incomplete packets (i.e., packets that present null features). In our experiments, we only remove the timestamp from the list since it does not illustrate the network behavior—instead, it is used for sorting. In this case, all other features are directly used to evaluate how different ML models perform in such circumstances.

These features are extracted based on proposals present in the literature regarding IoT security [8,46]. In fact, although these features have been used and validated in other efforts, our main goal is to present a flexible approach to training ML models with multiple features. Thus, several other features can be extracted or engineered based on the scripts used in this research as well as the raw network traffic (i.e., pcap files).

With the extracted features, we group the values captured in window sizes of 10 (i.e., Backdoor Malware, Benign Traffic, Browser Hijacking, Command Injection, Dictionary brute force, DNS spoofing, MITM ARP spoofing, Host Discovery, OS Scan, Ping Sweep, Port Scan, SQL Injection, Uploading Attack, Vulnerability Scan, and XSS) and 100 (DDoS ACK Fragmentation, DDoS HTTP Flood, DDoS ICMP Flood, DDoS ICMP Fragmentation, DDoS PSHACK Flood, DDoS RSTFIN Flood, DDoS SlowLoris, DDoS SYN Flood, DDoS SynonymousIP Flood, DDoS TCP Flood, DDoS UDP Flood, DDoS UDP Fragmentation, DoS HTTP Flood, DoS SYN Flood, DoS TCP Flood, DoS UDP Flood, Mirai GREIP Flood, Mirai Greeth Flood, and Mirai UDPPlain) packets to mitigate data size discrepancy (e.g., DDoS and CommandInjection) and calculate their mean values using Pandas [94] and Numpy [95]. Finally, we combine all subfiles into a processed csv dataset using Pandas. Thereupon, the resulting csv datasets represent the combination of features of each data chunk.

Moreover, each attack conducted in this research presents different characteristics. For example, the network traffic generated by a DDoS attack tends to be larger than the network traffic generated by a spoofing attack. Indeed, these differences can also be observed in other features of the dataset. Table 4 lists all features provided in the dataset, which Table 5 presents the characteristics of these features. For each feature in the entire dataset, we present the mean, standard deviation (std), minimum (min), 25th percentile (25%), median (50%), 75th percentile (75%), and maximum (max) values.

## 5. Machine Learning (ML) Evaluation

**Figure 9:**
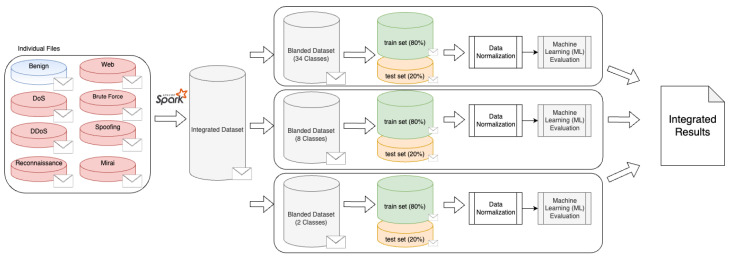
Machine learning (ML) evaluation pipeline adopted in this research.

In order to demonstrate how the CICIoT2023 dataset can be used to train machine learning (ML)-based attack detection and classification methods, Figure 9 illustrates the ML evaluation pipeline adopted in this research. Firstly, we combine all datasets produced following the procedure presented in Figure 8. In this sense, malicious and benign traffics are combined and shuffled into a single dataset (i.e., blended dataset) using PySpark [96]. Once the data are integrated, we evaluate ML performance from three different perspectives: (i) multiclass classification, focussing on classifying 33 individual attacks; (ii) grouped classification, considering 7 attack groups (e.g., DDoS and DoS); and (iii) binary classification (i.e., malicious and benign traffic classification). In each case, the dataset is divided into the train (80%) and test (20%) sets, which are normalized using the StandardScaler method [97] before the actual training process. Finally, the results obtained are summarized as integrated results.

### 5.1. Metrics

The evaluation of different ML models and configurations is conducted based on evaluation metrics. Given that *TP* represents the True Positives, *TN* the True Negatives, *FP* the False Positive, and *FN* the False Negatives, the metrics used in this research are [98]:

- **Accuracy:** responsible for evaluating the classification models by depicting the proportion of correct predictions in a given dataset and is based on the following expression:
  (1) $$Acc=\frac{TP+TN}{TP+TN+FP+FN}$$
- **Recall:** the ratio of correctly identified labels to the total number of occurrences of that particular label:
  (2) $$Rec=\frac{TP}{TP+FN}$$
- **Precision:** the ratio of correctly identified labels to the total number of positive classifications:
  (3) $$Pre=\frac{TP}{TP+FP}$$
- **F1-Score:** geometric average of precision and recall:
  (4) $$F1=2\times \frac{Pre\times Rec}{Pre+Rec}$$

### 5.2. Evaluation

**Figure 10:**
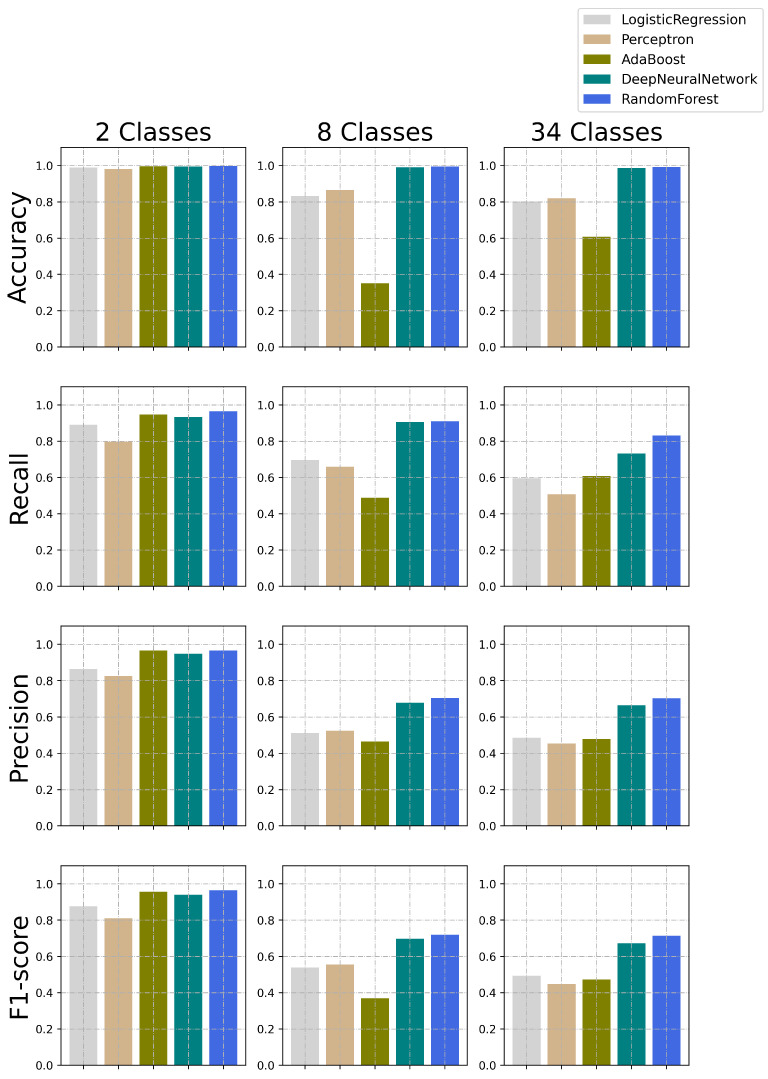
Results obtained in the classification process conducted using different machine learning models.

In the evaluation process, we adopted five ML methods that have been successfully used in different applications, including cybersecurity: Logistic Regression [99], Perceptron [100], Adaboost [101,102,103], Random Forest [104], and Deep Neural Network [105]. Figure 10 illustrates the performance of all methods when framing the classification problem as binary (i.e., malicious and benign), multiclass with 8 classes (i.e., benign and attack categories), and multiclass with 34 classes (i.e., benign and all individual attacks). These results are also depicted in Table 6.

For the binary classification, the results show that all methods present high performance, whereas accuracy is a metric that all methods reach over 98%, and the F1-score highlights the difference among these approaches. For example, Perceptron achieves 81%, showing that it suffers since the minority class (i.e., benign) is misclassified more often. In the classification of attack groups (i.e., eight classes), the overall performance is degraded since the classification task becomes more challenging. The Logistic Regression, Perceptron, and Adaboost methods show a significant decrease in accuracy. This impact is even more perceptible for F1-score. However, both Random Forest and Deep Neural Network are able to maintain high accuracy and F-1 score. These methods also present a decrease in performance but are capable of achieving F1 scores of 70%.

Finally, the most challenging classification task is represented by a multiclass classification of individual attacks (i.e., 34 classes). In this scenario, both Random Forest and Deep Neural Network could maintain high accuracy with very similar results. The same applies to F1-score since a slight reduction was perceived (around 1%) compared to the eight-class challenge. Furthermore, this case study shows that the Logistic Regression, Perceptron, and Adaboost methods are not able to categorize attacks as efficiently, given that the average accuracy is below 80% and F1-score is less than 50% in all cases.

These results show how ML methods can be used to classify attacks against IoT operations. In fact, this is a starting point that can be considered in any ML-based cybersecurity solutions for IoT operations. This effort not only highlights that the use of other ML methods is possible (e.g., optimized methods), but also enables the adoption of similar strategies to solve IoT-specific problems. Finally, although we are focussing on 33 different attacks, future directions could also be tailored to address issues related to individual attacks or categories.

### 5.3. Discussion

To illustrate how these models are performing for each class, Table 7 and Table 8 show the confusion matrix for Random Forest and Deep Neural Networks in the case of multiclass classification (eight classes).

In both cases, it is possible to observe that some classes are very well classified, mainly those with a large number of occurrences in the dataset. For example, the misclassification rates for DDoS, DoS, and Mirai are very small, followed by Recon and spoofing.

However, these models face challenges in classifying other attacks. For example, web-based attacks are usually classified as benign, Recon, or spoofing. The same occurs in the brute force classification. Although the similarities in the data patterns lead the models to make these mistakes, the classification is successful in most cases, leading to the results depicted in Figure 10. In fact, the results show that the multiclass classification performance degrades for three classes (Benign, Recon, and spoofing). The underlying traffic for those scenarios can be similar, and we intend to explore this phenomenon in future works further.

Finally, Table 9 and Table 10 compare all datasets reviewed with the proposed CICIoT2023 dataset. These tables focus on presenting an analysis of attacks executed in this research as well as its main contributions, i.e., these datasets may include attacks other than those shown in these tables.

## 6. Conclusions

Nowadays, IoT is becoming increasingly important for society. In this context, the development of security solutions is pivotal to enabling efficient, secure, and dependable IoT operations. This research introduced a novel and extensive IoT attack dataset to foster the development of security analytics applications in real IoT operations. In this process, 33 attacks are executed in an IoT topology composed of 105 devices. These attacks are classified into seven categories (i.e., DDoS, DoS, Recon, Web-based, brute force, spoofing, and Mirai) and all attacks are executed by malicious IoT devices targeting other IoT devices. Furthermore, this dataset includes multiple attacks not available in other IoT datasets and enables IoT professionals to develop new security analytics solutions using data in different formats. The dataset is available through the CIC Dataset website (https://www.unb.ca/cic/datasets/index.html, accessed on 19 June 2023).

Compared to the state-of-the-art publications, the CICIoT2023 dataset extends existing IoT security insights by using an extensive topology with a variety of IoT devices, executing several attacks never present in a single IoT security dataset, and analyzing how widely-used machine learning (ML) methods perform in different classification scenarios.

Finally, this work enables the development of several future works, e.g., the optimization of ML models, the analysis of features and how they influence different ML models, the interpretation of classifications, and the analysis of transferability based on the comparison to other datasets.

## Figures and Tables

**Table 1:** List of IoT devices used to produce the dataset.

	**Device Name**	**Category**	**MAC Address**	**Device Name**	**Category**	**MAC Address**
	Amazon Alexa Echo Dot 1	Audio	1C:FE:2B:98:16:DD	Lumiman bulb	Lighting	84:E3:42:42:ED:0B
	Amazon Alexa Echo Dot 2	Audio	A0:D0:DC:C4:08:FF	Philips Hue Bridge	Hub	00:17:88:60:D6:4F
	Amazon Alexa Echo Spot	Audio	1C:12:B0:9B:0C:EC	Smart Board	Home Automation	00:02:75:F6:E3:CB
	Amazon Alexa Echo Studio	Audio	08:7C:39:CE:6E:2A	Teckin Light Strip	Lighting	18:69:D8:EB:D4:3E
	Amazon Echo Show	Audio	2C:71:FF:05:F1:15	Teckin Plug 1	Power Outlet	D4:A6:51:76:06:64
	Google Nest Mini Speaker	Audio	CC:F4:11:9C:D0:00	Teckin Plug 2	Power Outlet	D4:A6:51:78:97:4E
	harman kardon (Ampak Technology)	Audio	B0:F1:EC:D3:E7:98	Wemo smart plug 1 (Wemo id: Wemo.Mini.AD3)	Power Outlet	30:23:03:F3:84:2B
	Sonos One Speaker	Audio	48:A6:B8:F9:1B:88	Wemo smart plug 2 (Wemo id: Wemo.Mini.4A3)	Power Outlet	30:23:03:F3:57:CB
	AMCREST WiFi Camera	Camera	9C:8E:CD:1D:AB:9F	Yutron Plug 1	Power Outlet	D4:A6:51:20:91:D1
	Arlo Base Station	Camera	3C:37:86:6F:B9:51	Yutron Plug 2	Power Outlet	D4:A6:51:21:6C:29
	Arlo Q Indoor Camera	Camera	40:5D:82:35:14:C8	LG Smart TV	Home Automation	AC:F1:08:4E:00:82
	Borun/Sichuan-AI Camera	Camera	C0:E7:BF:0A:79:D1	Netatmo Weather Station	Home Automation	70:EE:50:6B:A8:1A
	DCS8000LHA1 D-Link Mini Camera	Camera	B0:C5:54:59:2E:99	Raspberry Pi 4—2 GB	NextGen	DC:A6:32:C9:E6:F4
	HeimVision Smart WiFi Camera	Camera	44:01:BB:EC:10:4A	Raspberry Pi 4—2 GB	NextGen	DC:A6:32:C9: E4:C6
	Home Eye Camera	Camera	34:75:63:73:F3:36	Raspberry Pi 4—2 GB	NextGen	DC:A6:32:C9:E5:02
	Luohe Cam Dog	Camera	7C:A7:B0:CD:18:32	Fibaro Door/Window Sensor 1	Sensor	N/A
	Nest Indoor Camera	Camera	44:BB:3B:00:39:07	Fibaro Door/Window Sensor 2	Sensor	N/A
**Victms**	Netatmo Camera	Camera	70:EE:50:68:0E:32	Fibaro Door/Window Sensor 3	Sensor	N/A
	Rbcior Camera	Camera	10:5A:17:97:A5:C6	Fibaro Flood Sensor 1	Sensor	N/A
	SIMCAM 1S (AMPAKTec)	Camera	10:2C:6B:1B:43:BE	Fibaro Flood Sensor 2	Sensor	N/A
	TP-Link Tapo Camera	Camera	6C:5A:B0:44:1D:90	Fibaro Motion Sensor 1	Sensor	N/A
	Wyze Camera	Camera	7C:78:B2:86:0D:81	Fibaro Motion Sensor 2	Sensor	N/A
	Yi Indoor Camera	Camera	84:7A:B6:64:62:58	Fibaro Motion Sensor 3	Sensor	N/A
	Yi Indoor 2 Camera	Camera	84:7A:B6:62:3A:6C	Fibaro Motion Sensor 4	Sensor	N/A
	Yi Outdoor Camera	Camera	2C:D2:6B:66:D2:87	Fibaro Motion Sensor 5	Sensor	N/A
	Eufy HomeBase 2	Hub	8C:85:80:6C:B6:47	Fibaro Wall Plug 1	Power Outlet	N/A
	Amazon Plug	Power Outlet	B8:5F:98:D0:76:E6	Fibaro Wall Plug 2	Power Outlet	N/A
	Atomi Coffee Maker	Home Automation	68:57:2D:56:AC:47	Ring Alarm Keypad	Home Automation	N/A
	Cocoon Smart HVAC Fan	Home Automation	08:3A:F2:1F:BC:68	Ring Range Extender	Home Automation	N/A
	Globe Lamp ESP_B1680C	Lighting	50:02:91:B1:68:0C	Ring Contact Sensor (1)	Sensor	N/A
	GoSund Bulb	Lighting	C4:DD:57:13:07:C6	Ring Contact Sensor (2)	Sensor	N/A
	Gosund Power strip (1)	Power Outlet	50:02:91:1A:CE:E1	AeoTec TriSensor	Sensor	N/A
	GoSund Power strip (2)	Power Outlet	B8:F0:09:03:9A:AF	AeoTec Doorbell 6	Home Automation	N/A
	GoSund Smart plug WP2 (1)	Power Outlet	B8:F0:09:03:29:79	AeoTec Indoor Siren	Home Automation	N/A
	GoSund Smart Plug WP2 (2)	Power Outlet	50:02:91:10:AC:D8	AeoTec Smart Switch 7	Home Automation	N/A
	GoSund Smart plug WP2 (3)	Power Outlet	50:02:91:10:09:8F	AeoTec Water Sensor 6	Sensor	N/A
	GoSund Smart Plug WP3 (1)	Power Outlet	C4:DD:57:0C:39:94	AeoTec NanoMote Quad	Home Automation	N/A
	Gosund Smart Plug WP3 (2)	Power Outlet	24:A1:60:14:7F:F9	AeoTec Door/Window Sensor 7 Pro	Sensor	N/A
	Govee Smart Humidifer	Home Automation	D4:AD:FC:29:C8:A2	AeoTec Temperature and Humidity Sensor	Sensor	N/A
	HeimVision SmartLife Radio/Lamp	Lighting	D4:A6:51:30:64:B7	Philips Hue White 1	Lighting	N/A
	iRobot Roomba	Home Automation	50:14:79:37:80:18	Philips Hue White 2	Lighting	N/A
	LampUX RGB	Lighting	F4:CF:A2:34:48:6B	SmartThings Smart Bulb 1	Lighting	N/A
	Levoit Air Purifier	Home Automation	1C:9D:C2:8C:9A:94	SmartThings Smart Bulb 2	Lighting	N/A
	LIFX Lightbulb	Lighting	D0:73:D5:35:FB:C8	Aeotec Button	Home Automation	N/A
	SmartThings Hub	Hub	28:6D:97:7A:2B:2D	AeoTec Motion Sensor	Sensor	N/A
	AeoTec Smart Home Hub	Hub	28:6D:97:9E:F4:D5	AeoTec Multipurpose Sensor	Sensor	N/A
	Sengled Smart Plug 2	Power Outlet	N/A	AeoTec Water Leak Sensor	Sensor	N/A
	SmartThings Button	Home Automation	N/A	Sengled Smart Plug 1	Power Outlet	N/A
	SmartThings Smart Bulb 3	Lighting	N/A	Sonoff Smart Plug 2	Power Outlet	N/A
	Sonoff Smart Plug 1	Power Outlet	N/A	Arlo Ultra 2 Outdoor Camera	Camera	N/A
	Raspberry Pi 4—4 GB	NextGen	E4:5F:01:55:90:C4	Raspberry Pi 4—2 GB	NextGen	DC:A6:32:C9:E4:D5
	Raspberry Pi 4—8 GB	NextGen	DC:A6:32:DC:27:D5	Raspberry Pi 4—2 GB	NextGen	DC:A6:32:C9:E5:EF
**Attackers**	Raspberry Pi 4—2 GB	NextGen	DC:A6:32:C9:E4:AB	Raspberry Pi 4—2 GB	NextGen	DC:A6:32:C9:E4:90
	Raspberry Pi 4—2 GB	NextGen	DC:A6:32:C9:E5:A4	Ring Base Station	Hub	B0:09:DA:3E:82:6C
	Fibaro Home Center Lite	Hub	AC:17:02:05:34:27	Eufy Doorbell Camera	Camera	N/A

**Table 2:** CICIoT2023: tools and frameworks used to execute attacks.

	**Attack**	**Rows**	**Tool**
	ACK Fragmentation	285,104	hping3 [49]
	UDP Flood	5,412,287	udp-flood [50]
	SlowLoris	23,426	slowloris [51]
	ICMP Flood	7,200,504	hping3 [49]
	RSTFIN Flood	4,045,285	hping3 [49]
	PSHACK Flood	4,094,755	hping3 [49]
**DDoS**	HTTP Flood	28,790	golang-httpflood [52]
	UDP Fragmentation	286,925	udp-flood [50]
	ICMP Fragmentation	452,489	hping3 [49]
	TCP Flood	4,497,667	hping3 [49]
	SYN Flood	4,059,190	hping3 [49]
	SynonymousIP Flood	3,598,138	hping3 [49]
	TCP Flood	2,671,445	hping3 [49]
	HTTP Flood	71,864	golang-httpflood [52]
**DoS**	SYN Flood	2,028,834	hping3 [49]
	UDP Flood	3,318,595	hping3 [49] and udp-flood [50]
	Ping Sweep	2262	nmap [53] and fping [54]
	OS Scan	98,259	nmap [53]
**Recon**	Vulnerability Scan	37,382	nmap [53] and vulscan [55]
	Port Scan	82,284	nmap [53]
	Host Discovery	134,378	nmap [53]
	Sql Injection	5245	DVWA [56]
	Command Injection	5409	DVWA [56]
	Backdoor Malware	3218	DVWA [56] and Remot3d [57]
**Web-Based**	Uploading Attack	1252	DVWA [56]
	XSS	3846	DVWA [56]
	Browser Hijacking	5859	Beef [58]
**Brute Force**	Dictionary Brute Force	13,064	nmap [53] and hydra [59]
**Spoofing**	Arp Spoofing	307,593	ettercap [60]
	DNS Spoofing	178,911	ettercap [60]
	GREIP Flood	751,682	Adapted Mirai Source Code [61]
**Mirai**	Greeth Flood	991,866	Adapted Mirai Source Code [61]
	UDPPlain	890,576	Adapted Mirai Source Code [61]

**Table 3:** Number of rows for each attack and category.

Attack	Rows	Attack	Rows	Category	Rows
DDoS-ICMP_Flood	7,200,504	DoS-TCP_Flood	2,671,445	DDoS	33,984,560
DDoS-UDP_Flood	5,412,287	DoS-SYN_Flood	2,028,834	DoS	8,090,738
DDoS-TCP_Flood	4,497,667	BenignTraffic	1,098,195	Mirai	2,634,124
DDoS-PSHACK_Flood	4,094,755	Mirai-greeth_flood	991,866	Benign	1,098,195
DDoS-SYN_Flood	4,059,190	Mirai-udpplain	890,576	Spoofing	486,504
DDoS-RSTFINFlood	4,045,285	Mirai-greip_flood	751,682	Recon	354,565
DDoS-SynonymousIP_Flood	3,598,138	DDoS-ICMP_Fragmentation	452,489	Web	24,829
DoS-UDP_Flood	3,318,595	MITM-ArpSpoofing	307,593	BruteForce	13,064
Recon-PingSweep	2262	Uploading_Attack	1252		
DDoS-UDP_Fragmentation	286,925	DDoS-HTTP_Flood	28,790		
DDoS-ACK_Fragmentation	285,104	DDoS-SlowLoris	23,426		
DNS_Spoofing	178,911	DictionaryBruteForce	13,064		
Recon-HostDiscovery	134,378	BrowserHijacking	5859		
Recon-OSScan	98,259	CommandInjection	5409		
Recon-PortScan	82,284	SqlInjection	5245		
DoS-HTTP_Flood	71,864	XSS	3846		
VulnerabilityScan	37,382	Backdoor_Malware	3218		

**Table 4:** Features extracted from the network traffic.

#	Feature	Description
1	ts	Timestamp
2	flow duration	Duration of the packet’s flow
3	Header Length	Header Length
4	Protocol Type	IP, UDP, TCP, IGMP, ICMP, Unknown (Integers)
5	Duration	Time-to-Live (ttl)
6	Rate	Rate of packet transmission in a flow
7	Srate	Rate of outbound packets transmission in a flow
8	Drate,	Rate of inbound packets transmission in a flow
9	fin flag number	Fin flag value
10	syn flag number	Syn flag value
11	rst flag number	Rst flag value
12	psh flag numbe	Psh flag value
13	ack flag number	Ack flag value
14	ece flag numbe	Ece flag value
15	cwr flag number	Cwr flag value
16	ack count	Number of packets with ack flag set in the same flow
17	syn count	Number of packets with syn flag set in the same flow
18	fin count	Number of packets with fin flag set in the same flow
19	urg coun	Number of packets with urg flag set in the same flow
20	rst count	Number of packets with rst flag set in the same flow
21	HTTP	Indicates if the application layer protocol is HTTP
22	HTTPS	Indicates if the application layer protocol is HTTPS
23	DNS	Indicates if the application layer protocol is DNS
24	Telnet	Indicates if the application layer protocol is Telnet
25	SMTP	Indicates if the application layer protocol is SMTP
26	SSH	Indicates if the application layer protocol is SSH
27	IRC	Indicates if the application layer protocol is IRC
28	TCP	Indicates if the transport layer protocol is TCP
29	UDP	Indicates if the transport layer protocol is UDP
30	DHCP	Indicates if the application layer protocol is DHCP
31	ARP	Indicates if the link layer protocol is ARP
32	ICMP	Indicates if the network layer protocol is ICMP
33	IPv	Indicates if the network layer protocol is IP
34	LLC	Indicates if the link layer protocol is LLC
35	Tot sum	Summation of packets lengths in flow
36	Min	Minimum packet length in the flow
37	Max	Maximumpacket length in the flow
38	AVG	Average packet length in the flow
39	Std	Standard deviation of packet length in the flow
40	Tot size	Packet’s length
41	IAT	The time difference with the previous packet
42	Number	The number of packets in the flow
43	Magnitude	(Average of the lengths of incoming packets in the flow + average of the lengths of outgoing packets in the flow)${}^{0.5}$
44	Radius	(Variance of the lengths of incoming packets in the flow + variance of the lengths of outgoing packets in the flow)${}^{0.5}$
45	Covariance	Covariance of the lengths of incoming and outgoing packets
46	Variance	Variance of the lengths of incoming packets in the flow/ variance of the lengths of outgoing packets in the flow
47	Weight	Number of incoming packets × Number of outgoing packets

**Table 5:** Dataset description.

Feature	Mean	Std	Min	25%	50%	75%	Max
**flow_duration**	5.76544939	285.034171	0	0	0	0.10513809	394,357.207
**Header_Length**	76,705.9637	461,331.747	0	54	54	280.555	9,907,147.75
**Protocol Type**	9.06568989	8.94553292	0	6	6	14.33	47
**Duration**	66.3507169	14.0191881	0	64	64	64	255
**Rate**	9064.05724	99,562.4906	0	2.09185589	15.7542308	117.384754	8,388,608
**Srate**	9064.05724	99,562.4906	0	2.09185589	15.7542308	117.384754	8,388,608
**Drate**	5.46 × 10${}^{-6}$	0.00725077	0	0	0	0	29.7152249
**fin_flag_number**	0.08657207	0.28120696	0	0	0	0	1
**syn_flag_number**	0.20733528	0.40539779	0	0	0	0	1
**rst_flag_number**	0.09050473	0.28690351	0	0	0	0	1
**psh_flag_number**	0.08775006	0.28293106	0	0	0	0	1
**ack_flag_number**	0.12343168	0.32893207	0	0	0	0	1
**ece_flag_number**	1.48 × 10${}^{-6}$	0.00121571	0	0	0	0	1
**cwr_flag_number**	7.28 × 10${}^{-7}$	0.00085338	0	0	0	0	1
**ack_count**	0.09054283	0.28643144	0	0	0	0	7.7
**syn_count**	0.33035785	0.6635354	0	0	0	0.06	12.87
**fin_count**	0.09907672	0.32711642	0	0	0	0	248.32
**urg_count**	6.23982356	71.8524536	0	0	0	0	4401.7
**rst_count**	38.4681213	325.384658	0	0	0	0.01	9613
**HTTP**	0.04823423	0.21426079	0	0	0	0	1
**HTTPS**	0.05509922	0.22817383	0	0	0	0	1
**DNS**	0.00013068	0.01143079	0	0	0	0	1
**Telnet**	2.14 × 10${}^{-8}$	0.00014635	0	0	0	0	1
**SMTP**	6.43 × 10${}^{-8}$	0.00025349	0	0	0	0	1
**SSH**	4.09 × 10${}^{-5}$	0.00639772	0	0	0	0	1
**IRC**	1.50 × 10${}^{-7}$	0.00038722	0	0	0	0	1
**TCP**	0.57383427	0.49451846	0	0	1	1	1
**UDP**	0.21191758	0.40866676	0	0	0	0	1
**DHCP**	1.71 × 10${}^{-6}$	0.00130903	0	0	0	0	1
**ARP**	6.62 × 10${}^{-5}$	0.00813521	0	0	0	0	1
**ICMP**	0.16372157	0.37002273	0	0	0	0	1
**IPv**	0.99988731	0.01061485	0	1	1	1	1
**LLC**	0.99988731	0.01061485	0	1	1	1	1
**Tot sum**	1308.32257	2613.30273	42	525	567	567.54	127,335.8
**Min**	91.6073456	139.695326	42	50	54	54	13,583
**Max**	181.963418	524.030902	42	50	54	55.26	49,014
**AVG**	124.668815	240.991485	42	50	54	54.0497296	13,583
**Std**	33.3248065	160.335722	0	0	0	0.37190955	12,385.2391
**Tot size**	124.691567	241.549341	42	50	54	54.06	135,83
**IAT**	83,182,525.9	17,047,351.7	0	83,071,566	83,124,522.4	83,343,908	167,639,436
**Number**	9.49848933	0.81915318	1	9.5	9.5	9.5	15
**Magnitue**	13.12182	8.62857895	9.16515139	10	10.3923048	10.3967148	164.821115
**Radius**	47.0949848	226.769647	0	0	0	0.50592128	17,551.2708
**Covariance**	30,724.3565	323,710.68	0	0	0	1.34421569	154,902,159
**Variance**	0.0964376	0.233001	0	0	0	0.08	1
**Weight**	141.51237	21.0683073	1	141.55	141.55	141.55	244.6

**Table 6:** Results obtained in the classification process conducted using different machine learning models (illustrated in Figure 10).

	Metric	Logistic Regression	Perceptron	Adaboost	Random Forest (RF)	Deep Neural Network (DNN)
**34 classes**	**Accuracy**	0.80231507	0.8195961	0.607888	0.99164365	0.986118011
	**Recall**	0.59520185	0.507506	0.607675	0.831586401	0.731868794
	**Precision**	0.486752461	0.454634	0.479621	0.704492066	0.665295126
	**F1-score**	0.49388408	0.4472933	0.473498	0.714021981	0.672346883
**8 classes**	**Accuracy**	0.831674188	0.8663152	0.351357	0.994368173	0.991147043
	**Recall**	0.696055597	0.6591315	0.487789	0.91001105	0.906642708
	**Precision**	0.512409686	0.5239188	0.464924	0.705407564	0.679434746
	**F1-score**	0.539424048	0.5551339	0.368663	0.71928904	0.69726491
**2 classes**	**Accuracy**	0.989023188	0.9817525	0.995899	0.99680798	0.994422814
	**Recall**	0.890400624	0.7970288	0.947303	0.965163906	0.933277496
	**Precision**	0.863157959	0.825432	0.965631	0.965395244	0.947579486
	**F1-score**	0.876258983	0.8105374	0.956273	0.965279544	0.940305998

**Table 7:** Confusion matrix for Deep Neural Network in the case of multiclass classification (8 classes).

	Benign	Brute Force	DDoS	DoS	Mirai	Recon	Spoofing	Web
**Benign**	**230,229**	1	7	2	0	9270	3812	1
**Brute Force**	1054	**438**	3	0	0	1216	271	1
**DDoS**	23	0	**7,523,853**	1012	545	653	65	0
**DoS**	15	0	4933	**1,787,065**	60	61	33	0
**Mirai**	10	0	258	41	**583,283**	64	21	0
**Recon**	18,517	2	968	30	1	**55,656**	3455	1
**Spoofing**	30,485	0	17	0	15	10,021	**67,257**	3
**Web**	1976	0	1	0	0	2028	1221	**207**

**Table 8:** Confusion matrix for Random Forest in the case of multiclass classification (8 classes).

	Benign	Brute Force	DDoS	DoS	Mirai	Recon	Spoofing	Web
**Benign**	**234,929**	4	24	2	4	3192	5159	8
**Brute Force**	1342	**169**	1	0	0	844	626	1
**DDoS**	15	0	**7,525,049**	557	18	339	173	0
**DoS**	7	0	1088	**1,790,979**	34	12	47	0
**Mirai**	5	0	603	18	**582,921**	100	30	0
**Recon**	11,565	6	1418	11	16	**60,006**	5591	17
**Spoofing**	14,618	1	18	6	11	4743	**88,371**	30
**Web**	1140	1	3	1	1	1265	2792	**230**

**Table 9:** Comparison CICIoT2023 with existing IoT security datasets.

	Attack	IoTHIDS	N-BaIoT	Kitsune	IoTNIDS	IoT-SH	BoT-IoT	MedBIoT	IoT-23 (2020)	IoTIDS	MQTT	MQTT-IoT-IDS	X-IIoTID	WUSTL-IIoT	Edge-IIoTSet	CICIoT2023
	ACK Fragmentation	-	-	-	-	-	-	-	-	-	-	-	-	-	-	**✓**
	UDP Flood	-	✓	-	✓	-	✓	-	-	-	-	-	-	-	✓	**✓**
	SlowLoris	-	-	-	-	-	-	-	-	-	-	-	-	-	-	**✓**
	ICMP Flood	-	-	-	-	-	-	-	-	-	-	-	-	-	✓	**✓**
	RSTFIN Flood	-	-	-	-	-	-	-	-	-	-	-	-	-	-	**✓**
	PSHACK Flood	-	-	-	-	-	-	-	-	-	-	-	-	-	-	**✓**
	HTTP Flood	-	✓	-	✓	-	✓	-	-	-	-	-	-	-	✓	**✓**
**DDoS**	UDP Fragmentation	-	-	-	-	-	-	-	-	-	-	-	-	-	-	**✓**
	ICMP Fragmentation	-	-	-	-	-	-	-	-	-	-	-	-	-	-	**✓**
	TCP Flood	-	✓	-	-	-	-	-	-	-	-	-	-	-	✓	**✓**
	SYN Flood	-	✓	-	-	-	-	-	-	-	-	-	-	-	✓	**✓**
	SynonymousIP Flood	-	-	-	-	-	-	-	-	-	-	-	-	-	-	**✓**
	TCP Flood	-	✓			✓	✓	-	-	-	-	-	-	-	-	**✓**
**DoS**	HTTP Flood	-	✓	-	-	-	✓	-	-	✓	-	-	-	-	-	**✓**
	SYN Flood	-	✓	✓	✓	-	-	-	-	✓	-	-	-	-	-	**✓**
	UDP Flood	-	✓	-	-	✓	✓	-	-	✓	-	-	-	-	-	**✓**
	Ping Sweep	-	-	-	-	-	-	-	-	-	-	-	-	-	-	**✓**
	OS Scan	-	-	✓	✓	✓	✓	-	-	✓	-	✓	✓	-	✓	**✓**
**Recon**	Vulnerability Scan	-	✓	-	-	-	-	-	-	-	-	✓	✓	-	✓	**✓**
	Port Scan	-	✓	-	✓	✓	✓	-	-	✓	-	✓	✓	-	✓	**✓**
	Host Discovery	-	-	-	✓	-	-	-	-	-	-	-	✓	-	-	**✓**
	Sql Injection	-	-	-	-	-	-	-	-	-	-		-	-	✓	**✓**
	Command Injection	-	-	-	-	-	-	-	-	-	-	-	-	-	-	**✓**
	Backdoor Malware	-	-	-	-	-	-	-	-	-	-	-	✓	-	✓	**✓**
**Web-Based**	Uploading Attack	-	-	-	-	-	-	-	-	-	-	-	-	-	✓	**✓**
	XSS	-	-	-	-	-	-	-	-	-	-		-	-	✓	**✓**
	Browser Hijacking	-	-	-	-	-	-	-	-	-	-	-	-	-	-	**✓**
**Brute** **Force**	Dictionary Brute Force	-	-	-	✓	-	-	-	-	-	✓	✓	✓	-	✓	**✓**
**Spoofing**	Arp Spoofing	-	-	✓	✓	✓				✓	-	-	✓		✓	**✓**
	DNS Spoofing	-	-	-	-	✓	-	-	-	-	-	-	-	-	✓	**✓**
	GREIP Flood	✓	✓	✓	✓	-	-	✓	✓	✓	-	-			-	**✓**
**Mirai**	Greeth Flood	✓	✓	✓	✓	-	-	✓	✓	✓	-	-	-	-	-	**✓**
	UDPPlain	✓	✓	✓	✓	-	-	✓	✓	✓	-	-	-	-	-	**✓**

**Table 10:** Comparison CICIoT2023 contributions with existing IoT security datasets.

	Extensive Topology (>100 Devices)	Execution of 33 Attacks Divided into 7 Classes	Machine Learning and Deep Learning Evaluation
**IoTHIDS**			
**N-BaIoT**			✓
**Kitsune**			✓
**IoTNIDS**			✓
**IoT-SH**			✓
**BoT-IoT**			✓
**MedBIoT**			✓
**IoT-23 (2020)**			✓
**IoTIDS**			✓
**MQTT**			✓
**MQTT-IoT-IDS**			✓
**X-IIoTID**			✓
**WUSTL-IIoT**			✓
**Edge-IIoTSet**			✓
**CICIoT2023**	**✓**	**✓**	**✓**

## Data Availability

https://www.unb.ca/cic/datasets/iotdataset-2023.html, accessed on 19 June 2023.
